# Disentangling Trait-Like Between-Individual vs. State-Like Within-Individual Effects in Studying the Mechanisms of Change in CBT

**DOI:** 10.3389/fpsyt.2020.609585

**Published:** 2021-01-21

**Authors:** Sigal Zilcha-Mano, Christian A. Webb

**Affiliations:** ^1^Department of Psychology, University of Haifa, Haifa, Israel; ^2^McLean Hospital and Harvard Medical School, Boston, MA, United States

**Keywords:** personalized treatment, mechanisms of change, process of change, between-individual effects, within-individual effect, State-like, Trait-like

## Abstract

Hofmann et al. argued that “[w]hile the clinical field has produced a dizzying number of treatment models and treatment protocols for virtually every psychiatric and psychological problem imaginable, increases in understanding of the processes of change in psychotherapy has been slow to arrive.” We propose that one of the reasons for the slow progress is that prior psychotherapy research conflates trait-like and state-like components of mechanisms of change. Trait-like components can serve as prescriptive or prognostic variables, whereas state-like components reflect within-client processes of change, and may highlight active ingredients of successful treatment. Distinguishing between the two is essential for clarifying the underlying processes of change in psychotherapy, and ultimately identifying empirically-derived individualized treatment targets. We review studies that implement methodological and statistical approaches for disentangling the two. These studies clarified particular mechanisms of change that may operate in a given treatment, highlighted differences in the processes of change between different treatments, and explored the within-individual interplay between different mechanisms of change during treatment. Examples include studies investigating the therapeutic role of behavioral, cognitive, and interpersonal skills, as well as emotional processing. We conclude with suggestions for future research, including attention to diversity, improved measurement to facilitate a reliable and valid estimation of trait-like and state-like components, the use of appropriate statistical approaches to adequately disentangle the two components, integration of theory-driven and data-driven methods of analysis, and the need to experimentally manipulate the state-like changes in a given mechanism of change to strengthen causal inferences.

## Introduction

Theoretical conceptualizations of the mechanisms underlying psychotherapeutic change refer to dynamic, multivariable processes which unfold over the course of treatment (1). Within-client state-like changes in theory-specified mechanisms of change are assumed to contribute to reductions in symptoms and improvements in well-being. Researchers in many fields of science have shown that the trait-like qualities of a construct and state-like changes in it over time are meaningfully distinct entities and critical to disaggregate (2). One commonly used example illustrating the importance of disentangling trait-like and state-like components of the same construct is the association between typing speed (number of words typed per minute) and the percentage of typing errors made (3). At the between-individual or trait-like level, there is an inverse association: individuals who type faster tend to make fewer mistakes than those who type more slowly. In contrast, at the state-like level (i.e., within individuals), the association between typing speed and typos is positive: the faster one types, the more errors one is likely to make. As another example of the need to disentangle trait vs. state level effects, individuals who exercise more are, on average, at decreased risk of a heart attack relative to those who do not. However, at the individual level, one is at a higher risk of a heart attack during intensive exercise relative to at rest (4). A third example in which trait-like and state-like effects show opposite directions is of the effect of self-efficacy on performance. Whereas the trait-like effect of self-efficacy on performance is positive [individuals with higher self-efficacy show better performance; (5)], the state-like effect is negative [a state-like boost in self-efficacy may result in poorer performance; (6)], due, perhaps, to overconfidence in one's abilities.

As these three examples demonstrate, effects examined at the trait-like vs. state-like level can not only be inconsistent, but even opposite in direction. In addition, as described in more details below, trait-like level characteristics may moderate state-like effects (e.g., the within-individual association of exercise on heart attack risk is moderated by pre-existing cardiovascular risk factors). Below, we discuss the importance of disentangling trait-like and state-like effects to clarify the mechanism of change in CBT and for informing treatment selection and targets.

## Key Challenge in the Study of Mechanisms of Change in CBT: Conflating Trait-Like and State-Like Effects

Reviews and meta-analyses on the mechanisms of change in CBT for depression suggest that CBT may improve dysfunctional thinking (7), and in turn, that cognitive change is associated with better treatment outcomes (8). However, most studies on the mechanism of change in CBT, including those focused on the core question of the role of cognitive change, are constrained in the causal inferences they can draw due to methodological limitations and have yielded mixed findings (9). For example, in the treatment of depression, a meta-analysis suggested that adherence and competence are, on average, not significantly associated with treatment outcome, with mixed findings across the included studies (10). Mixed results have also been obtained for treatments of anxiety disorders. For example, Foa and Kozak's emotional processing theory (11) was supported by some studies (12, 13) but not by others (14, 15). Similarly, the inhibitory learning theory of Craske et al. (16) produced mixed results, with some studies supporting it (14, 17) and others describing a more complex picture (18). The mixed results are so profound that in their systematic review of the literature on common factors across psychotherapies, Cuijpers, Reijnders, and Huibers (19) concluded that: “It is as if we have been in the pilot phase of research for five decades without being able to dig deeper” (p. 224).

An important factor that may help account, at least in part, for the mixed results is that most studies conflate trait-like and state-like components. As others have emphasized, it is critical to disentangle trait-like (between-individuals variance) and state-like (within-individual variance) components (2, 20), especially with the type of data generated in psychotherapy research (21). Inferences drawn from studies that do not disaggregate trait-like and state-like components can be strikingly different relative to those that do. As has been argued by Fisher and colleagues (21): “… conclusions drawn from aggregated data may be worryingly imprecise” (p. 6106). Trait-like variability refers to any variance between individuals in their traits or relatively stable characteristics. For example, within psychotherapy, trait-like characteristics may describe relatively enduring, automatic pre-treatment patterns of thoughts, feelings, and behaviors that are consistent across similar situations. Trait-like components may refer to relatively fixed entities (like many demographic variables) or to a recurring, dynamic pattern that characterizes the individual [i.e., predicable diurnal cycles in anxiety; (22)]. State-like changes may include reductions or increases in a trait-like characteristic (e.g., reduction in previously stable levels of anxiety) or deviations from a previously stable dynamic pattern (e.g., attenuation of a strong diurnal pattern of anxiety), potentially as the result of treatment. The trait-like components may serve as (a) “prognostic” (i.e., treatment non-specific) predictors – stable client characteristics that influence one's ability to benefit from any treatment (e.g., cognitive impairment or interpersonal pathology) or as (b) “prescriptive” variables (i.e., moderators) – variables that predict differential response to one treatment vs. another (e.g., CBT vs. antidepressants). In contrast, state-like components refer to within-individual variation in a construct that occur over time, such as in a mechanism of change as a result of implementing therapeutic techniques that target those mechanisms. State-like changes in those mechanisms are in turn expected to bring about changes in symptoms. The trait-like vs. state-like distinction may shed light on inconsistent earlier findings.

## Benefits of Disaggregating Trait-Like and State-Like Components

### Clarifying the Mechanisms of Change

**Figure 1:**
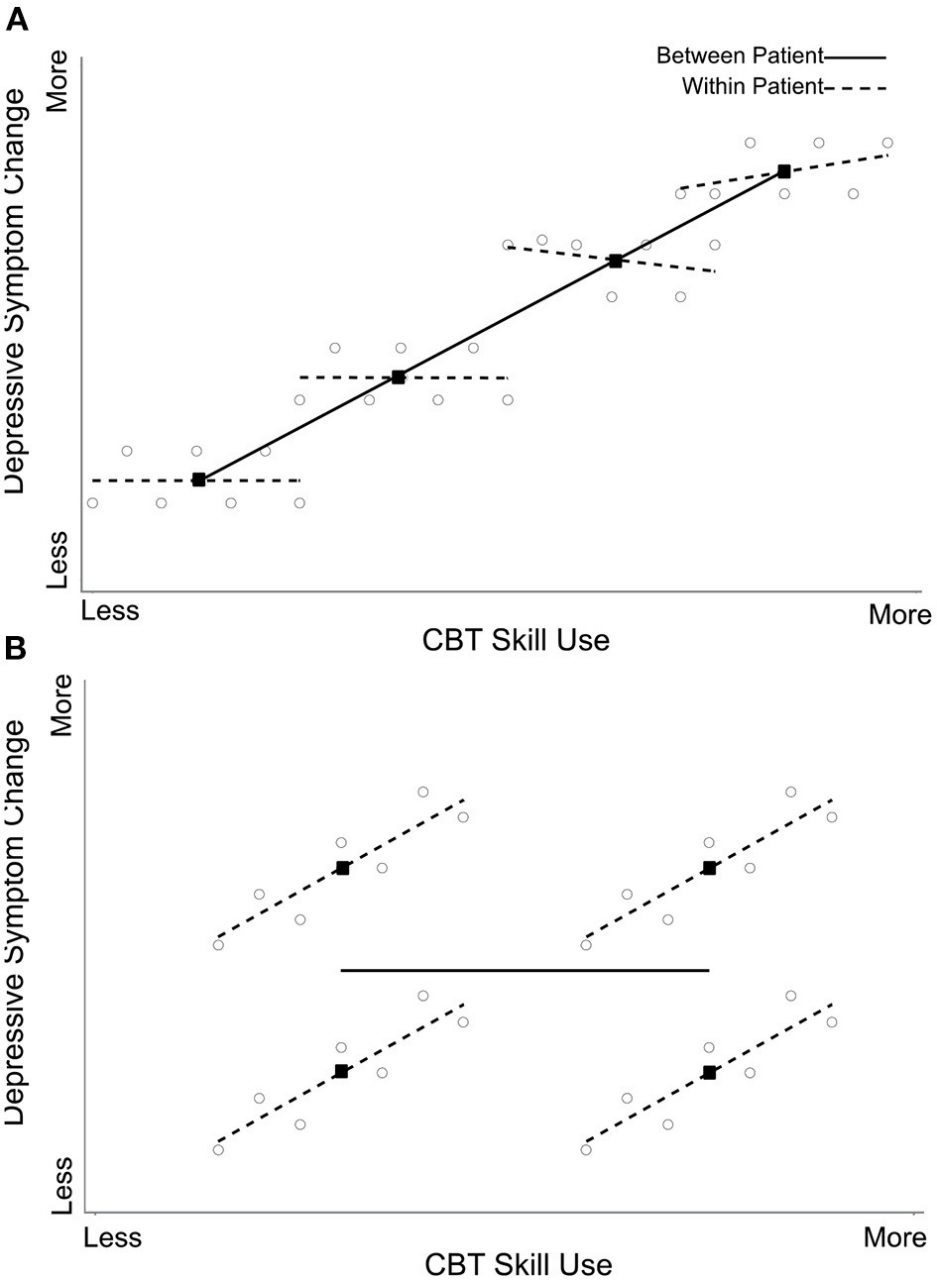
Webb et al. (23) American Psychological Association. Reprinted with permission. The figure displays simulated data from four clients (seven time points per client) showing a between-client (dark line), but no within-client (dotted lines), effect of skills on symptom improvement **(A)** vs. a within-client, but no between-client, effect of skills on outcome **(B)**. Circles represent skill scores for each client at each time points, and black squares refer to each client's mean skill score.

A core feature of CBT is the focus on the acquisition of cognitive (e.g., identifying and interrogating negative automatic thoughts) and behavioral (e.g., behavioral activation) skills. To what extent does client use of cognitive and/or behavioral skills in fact contribute to depressive symptom change? To adequately address this question, the state-like component (i.e., variance in cognitive and behavioral skills *within* clients over the course of treatment) needs to be isolated from the trait-like component (i.e., stable, between-client differences in the tendency to report generally high vs. low levels of cognitive or behavioral skills) (see Figure 1 for a simulated example to illustrate state-like vs. trait-like effects). In a recent study, Webb et al. (23) found that client-reported use of behavioral – but not cognitive – skills predicted symptom change in CBT for depressed adolescents. The latter finding emerged when using conventional analyses (i.e., not disaggregating state-like and trait-like components). However, when disaggregating these two components, only the state-like components were significant. Specifically, and consistent with a causal interpretation, greater state-like within-individual levels of behavioral skills predicted greater depressive symptom change. The same pattern of findings emerged whether client skills were assessed from the perspective of the client themselves or from the therapist. Importantly, and similar to the abovementioned typing speed and heart attack examples, one could certainly imagine how trait-like and state-like effects could operate in opposite directions. For example, those individuals with trait-like deficits in behavioral activation (BA) skills may be more likely than those with relatively high levels of skills to benefit from BA therapy, given that the latter treatment specifically targets that skill set (i.e., *lower* trait-like skills predicts relatively enhanced response to BA). In contrast, greater state-like within-individual increases in BA skills may predict better outcomes within treatment (i.e., the opposite relation for the state-like effect). An example for such opposite directions of trait-like and state-like components comes from a study by Rubel and colleagues (24). In their study, higher levels of state-like in-session affective experiences and involvement were associated with a greater subsequent reduction in symptoms. However, the trait-like effects were in the opposite direction: higher overall levels of affective experiences were associated with higher overall symptom severity.

Disentangling trait-like and state-like components is also important for identifying which techniques bring about changes within individuals. For example, when using conventional analyses (i.e., not disentangling trait-like and state-like components), both adherence to identifying and evaluating automatic thoughts and adherence to negotiating therapy content with the client and structuring the session were significant predictors of treatment outcome (25). In contrast, with the trait-like vs. state-like distinction, only state-like changes in adherence to identifying and evaluating automatic thoughts predicted next-session symptom change. Such findings may help inform which techniques therapists should consider implementing in a session to bring about better treatment outcomes (26). Another example for distinct effects at the trait-like and state-like levels comes from the research on non-verbal synchrony. Recent findings suggest that at the between-individual level, trait-like non-verbal synchrony was not associated with either problem actuation or motivational clarification. However, at the within-client level, state-like non-verbal synchrony was associated with both problem actuation and motivational clarification (27).

### Identifying Differences in Mechanisms of Change Between Treatments

One of the most replicated findings in psychotherapy research is that treatments conceptualized as working via different mechanisms often show similar outcomes at the end of treatment [commonly referred to as the *Dodo Bird Verdict*, (28, 29)]. Based on this finding, many scholars have argued that all treatments work through the same mechanisms, and consequently questioned the claim that different treatments have unique mechanisms of change. We argue that the trait-like vs. state-like distinction may have the potential to reveal different mechanisms of change underlying distinct treatments. Of relevance, a recent study (30) comparing exposure-based cognitive therapy (EBCT) and cognitive-behavioral therapy (CBT) for depression found that although EBCT augments CBT by the addition of exposure-based strategies, no significant differences between the two conditions emerged in their treatment outcomes. After making the trait-like vs. state-like distinction (using a centering approach), however, EBCT was found to result in greater state-like increases in emotional processing during treatment and higher self-efficacy during follow-up relative to CBT, both of which were associated with better long-term depression outcome (31). One potentially fruitful avenue for future research is to identify individuals who may benefit most from integrating emotional processing strategies. For those individuals, EBCT may result in better outcomes than CBT, given that the former treatment directly targets emotional processing. Another example comes from research on the working alliance, which is commonly referred to as a non-specific common factor. Studies suggest that trait-like differences in the alliance between patients are indeed associated with treatment outcome across different treatments, with stronger alliances being linked to better outcomes (32). However, in treatments that directly focus on the alliance as a mechanism of change (e.g., brief relational treatment), SL changes in alliance were stronger predictors of subsequent treatment outcome, than in treatments where the alliance is typically not considered a main mechanism of change (33, 34).

### The Longitudinal Interplay Between Different Mechanisms of Change

Although studies commonly focus on a single mechanism of change, the reality of clinical practice teaches us that for a given individual a variety of factors – and complex interactions among them – are contributing to symptom change. For example, state-like changes in one mechanism may be moderated by trait-like levels of another, suggesting that the processes or mechanisms of change may differ as a function of identifiable client characteristics, and thus answering the question for whom a given therapeutic procedure may be most beneficial. For example, Fitzpatrick et al. (35) explored the question of who benefits most from cognitive change in cognitive therapy for depression. After disaggregating state-like and trait-like components of cognitive change, using a centering approach (20), the authors found that clients with poorer trait-like interpersonal skills and greater trait-like interpersonal problems exhibited a stronger relation between state-like changes in cognition and symptom improvement.

Moreover, state-like changes in one mechanism may be moderated by state-like changes in another. This type of interaction may suggest *how* two mechanisms of change interact to bring about therapeutic change. Interactions between state-like components of two or more mechanisms or other process variables may also guide clinical decisions on *when* to target a specific mechanism. Specifically, state-like changes in process variables may provide useful and actionable information about the optimal timing for implementing procedures that target a specific mechanism of change. In this case, the interaction may suggest *when* (i.e., at which levels of the process variable) state-like changes in a particular mechanism of change are most beneficial in bringing about therapeutic change. For example, Zilcha-Mano (36) found that state-like improvements in alliance at a given session result in subsequent reduction in symptoms only in the case of higher sense of life satisfaction at that session, thus suggesting when it may be most therapeutically beneficial to implement techniques for strengthening the alliance.

Interactions between trait-like and state-like components of the same construct may be of particular interest, because they may contribute to progress toward precision medicine (37). Such interactions serve as a test of two contrasting hypotheses: building on clients' relative weaknesses vs. capitalizing on their strengths (38). A recent meta-analysis based on individual level data from 5,350 individuals suggested that the effect of state-like changes in alliance on outcome was stronger for individuals with stronger trait-like alliance (39). This finding supports the capitalizing on the clients' strengths hypothesis: those with stronger trait-like alliance are the ones who derive the most therapeutic benefit from state-like gains in the alliance. Building on the BA example above, individuals with relatively higher baseline competency in BA skills may be more likely to take advantage of a BA treatment that capitalizes on their pre-existing strengths (40, 41). Whereas the latter example is consistent with a “capitalization” model, one could also imagine a “compensatory” model [i.e., individuals with trait-like deficits in BA skills benefit the most from a treatment (BA) that directly targets their deficit].

The examples so far focused on moderation. However, another way in which state-like changes in two variables can relate to each other is by one preceding the other, in a within-client mediation model, to delineate the *temporal process of how therapeutic change occurs*. For example, Schmidt et al. (42) found that immediate state-like cognitive changes predicted sustained cognitive changes, which in turn predicted treatment outcome.

## What Does the Future Hold?

Below we highlight several promising directions for future treatment research disentangling state-like within-person vs. trait-like between-person effects.

### Better Measurement Will Facilitate a More Reliable and Valid Estimation of the Trait-Like and State-Like Components

**Figure 2:**
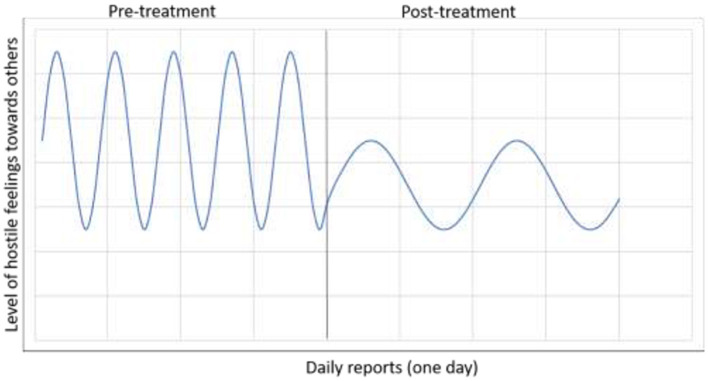
A demonstration of change in both the overall mean level of a construct and in its dynamic pattern as the result of treatment. The client started treatment with a generally high level of negative affect, and specifically, higher levels of hostile feelings toward others, characterized by a pattern of frequent and extreme daily reports of hostility. In the course of treatment, the overall mean level of hostile feelings was reduced. The dynamic pattern changed as well, the client displaying less frequent and less extreme hostility toward others.

When distinguishing between trait-like and state-like components, the ability to capture dynamic patterns is critical. Based on an accurate assessment of baseline trait-like dynamics, it is possible to investigate not only whether the individual's average values of a construct have changed, but also whether the trait-like dynamics have changed. For example, mean level of negative affect (NA) may change as a result of effective treatment, and the dynamic pattern of the individual may change as well (e.g., attenuated fluctuations in NA; Figure 2). Capturing this dynamic before, during the course of, and after treatment requires frequent sampling of NA in the daily lives of individuals. Given the omnipresence of smartphones, ecological momentary assessment (EMA) has become increasingly popular in psychological research and holds promise for psychotherapy studies investigating relevant state-like within-person vs. trait-like between-person processes. In addition to EMA, the expansion of “passive” (i.e., no user input required) measurement methods also holds promise for examining predictors and processes of change, including sensor data (e.g., activity levels and movement from accelerometer and GPS, proxies of social interaction from call and text meta-data) from smartphones and wearables (43), as well as other markers based on motion (44, 45), acoustic and language style (46–48) and physiology (49). The extent to which biological variables, such as hormones (50), neuroimaging (51–53) and inflammatory biomarkers (54), provide incremental predictive validity above conventional (and less costly and time-consuming) self-report measures is also an important area of research (55).

### The Use of Appropriate Statistical Analyses for Disentangling Trait-Like and State-Like Components

The trait-like vs. state-like distinction requires specialized statistical approaches to disaggregate and analyze these two components. First, it is important to use the appropriate methods to make the distinction, which fit the type of the data collected (20), and it is equally important to use appropriate statistical methods in analyzing each of the two components. Many statistical methods currently being used to analyze psychotherapy data are suitable for handling the trait-like components of mechanisms of change, but not the state-like components. For example, the analyses conducted to identify factors at the basis of the majority of available self-report scales are appropriate for trait-like components, but not for state-like components, and yet the same scales are often used to assess within-subject, should be within-individual. Factor structures of a scale for trait-like and state-like components of the same construct may differ (56). It is essential, therefore, to use the factor analyses that are suitable for state-like data. As another example, Group Iterative Multiple Model Estimation [GIMME; (57)], based on a unified structural equation modeling [uSEM; (58)] framework, integrates within-individual (idiographic) and conventional between-individual (nomothetic) modeling. Specifically, GIMME estimates subject-specific associations, as well searches for commonalities between individuals in those relations (59). With sufficient data points per individual (e.g., repeated EMA of relevant mechanism of change variables and outcome assessments), GIMME may allow psychotherapy researchers to estimate common patterns in mechanisms of change across clients, while simultaneously capturing individual-level heterogeneity in those variable relations (i.e., client-specific patterns). A detailed discussion of GIMME, and related approaches, is beyond the scope of this review. However, it is important to note that there are a number of assumptions that should be met with such time series data (e.g., stationarity, approximately equal time intervals between assessments, continuous variables; see (59, 60).

### Integration of Theory-Driven and Data-Driven Methods of Analysis

Data-driven approaches have been increasingly common in psychotherapy research in recent years and may have fruitful applications for research focused on trait-like vs. state-like distinctions (40, 61, 62). As one example from relationship science, a team of researchers recently sought to predict the construct of relationship quality (63). Using a machine learning approach with a total sample of 11,196 and 2,413 potential predictors, the researchers discovered a similar pattern of findings to those that have accumulated in many fields of science: the trait-like and state-like components of relationship quality produce distinct patterns. Up to about half the variance in the trait-like component of relationship quality can be explained by the individual's baseline trait-like predictors (e.g., attachment avoidance). By contrast, the variance of the state-like component of relationship quality (that is, relationship quality change) was largely unpredictable. Similar results demonstrating the differences in predicting trait-like vs. state-like components were obtained regarding other constructs [e.g., (64–66)]. With regards to research investigating mechanisms of change in psychotherapy research, recent work has revealed the challenges in identifying predictors of the state-like effect of alliance on outcome (62), although some promising results have been obtained when a variety of potential interpersonal predictors were used (67). Promising results have also been obtained in a recent study using a machine learning approach to predict client-specific skill-affect associations based on baseline clinical and demographic characteristics (59). These preliminary findings on the implementation of machine leaning approaches to identifying predictors of state-like effects stress the importance of thoughtful selection of relevant predictors in future trial designs, as well as consideration of a variety of machine leaning-related analytical approaches. It is also worth noting that computational models of psychological change and recovery that attempt to directly emulate the psychological mechanisms occurring within each individual client may contribute to progress in psychotherapy research toward precision medicine (68).

### Demonstrating Causality

Establishing a correct temporal relationship between state-like changes in a mechanism of change and subsequent symptomatic change is important in progress toward inferring causality, but a more direct (experimental) test of the effect of state-like manipulation is needed. Examples of direct manipulation of mechanisms of change include the administration of D-cycloserine and hydrocortisone as facilitators of inhibitory learning in exposure therapy (18), as well as the direct modulation of brain function connectivity using approaches such as transcranial magnetic stimulation (TMS) of the cerebellar midline (69).

### Attention to Diversity

Different mechanisms of change may be at play for different populations. As Hollon (70) has argued, moderated mediation models can improve the precision of the tested mediation model because they take into account the different processes that may come into play for different individuals. Potential moderators may include clinical symptoms [e.g., therapists adherence to cognitive techniques may play a relatively more prominent role in contributing to symptom change among clients with more severe depressive symptoms, (71); whereas the reverse may be the case with regards to the alliance, (39)] and socio-demographic variables [e.g., adherence to cognitive techniques may be more critical for women than men (72)]. Attention to such diversity may contribute to more contextually appropriate implementations of therapeutic procedures to bring about state-like changes in specific mechanisms of change.

## Summary

Although studies focused on the mechanisms of change in psychotherapy have been published at a rapid rate, our understanding of underlying processes of change has made slow progress and produced contradictory results. In the present article, we propose that one contributing factor to the slow advance and the mixed results is the conflation of trait-like and state-like components of individual mechanisms of change. As has been demonstrated before, the two components have distinct meanings and play different roles in treatment (37), and studies can yield very different findings depending on whether these two components are conflated or disaggregated (21, 32).

As reviewed, studies leveraging methodological and statistical approaches to disaggregate trait-like and state-like components can yield important findings on the processes of psychotherapeutic change, including: (a) clarifying within-client mechanisms of change in CBT (as in the example of state-like changes in behavioral skills predicting a reduction in depressive symptoms, Figure 1); (b) identifying differences between treatments in putative mechanisms of change (as in the example of the mechanisms targeted in ECBT vs. CBT); and (c) exploring the interplay between mechanisms of change in the process of bringing about therapeutic change, with the aim of clarifying the optimal circumstances and timing for targeting any given mechanism or a series of mechanisms (such as interactions between trait-like and state-like components of multiple mechanisms of change to answer the questions for whom, when, and how to implement given therapeutic procedures). It is of course important to note that disaggregating state-like and trait-like effects is relevant to psychotherapy research more broadly, and not just CBT (37).

We are optimistic about the future of psychotherapy science implementing the trait-like vs. state-like distinction using interdisciplinary approaches. The accumulation of data making this distinction will be instrumental in building clear and detailed links between evidence-based procedures (e.g., exposure, mindfulness practices) and evidence-based mechanisms and processes (e.g., cognitive flexibility and diffusion/distancing)(1).

## Data Availability Statement

The original contributions presented in the study are included in the article/supplementary materials, further inquiries can be directed to the corresponding author/s.

## Author Contributions

SZ-M and CW contributed equality to conceiving the presented ideas and to the writing of the manuscript. All authors contributed to the article and approved the submitted version.

## Conflict of Interest

The authors declare that the research was conducted in the absence of any commercial or financial relationships that could be construed as a potential conflict of interest.
